# Miscibility, Morphology and Crystallization Behavior of Poly(Butylene Succinate-*co*-Butylene Adipate)/Poly(Vinyl Phenol)/Poly(l-Lactic Acid) Blends

**DOI:** 10.3390/polym8120421

**Published:** 2016-12-06

**Authors:** Pengfei Si, Faliang Luo, Fahai Luo

**Affiliations:** 1 Ministry-Province Co-Cultivated Key Laboratory Base of Natural Gas Conversion, Ningxia University, Yinchuan 750021, China; sipf_0107@163.com (P.S.); lfhnxlp@163.com (F.L.); 2 School of Chemistry and Chemical Engineering, Ningxia University, Yinchuan 750021, China; 3 Teaching and Research Group of Physics, Liupanshan High School of Ningxia, Yinchuan 750002, China

**Keywords:** poly(l-lactic acid), poly(butylene succinate-*co*-butylene adipate), miscibility, crystallization, hydrogen bonding

## Abstract

Amorphous poly(vinyl phenol) (PVPh) is introduced into poly(butylene succinate-*co*-butylene adipate)/poly(l-lactic acid) (PBSA/PLLA) blends via solution casting. Fourier transform infrared spectroscopy (FTIR) analysis verifies that intermolecular hydrogen bonding formed in PBSA/PVPh/PLLA blends. The miscibility between PBSA and PLLA is improved with PVPh incorporation as evidenced by approaching *T*_g_s of the two components. When PVPh content reaches up to 50 wt %, the blend sample exhibits only one *T*_g_, meaning complete miscibility between PBSA and PLLA. The improved miscibility of PBSA/PLLA blends is further confirmed by scanning electron microscope (SEM). Typical “see-island” phase separation structure for PBSA/PLLA blend transforms into homogenous phase structure for blend samples with 5 wt % PVPh and above. Non-isothermal crystallization analysis shows that the crystallization temperature and crystallization enthalpy of PBSA decrease with PVPh addition, and those of PLLA also show a decreasing trend. Isothermal crystallization rate of PBSA in blend samples distinctly decreases with PVPh incorporation, whereas that of PLLA in blend samples increases slightly with PVPh addition. Wide angle X-ray diffraction (WAXD) analysis indicated that PLLA in blend samples remained partly crystallized, while PBSA turned into amorphous state with increasing PVPh contents.

## 1. Introduction

The indiscriminate use of fossil-based plastics had produced severe environmental problems, which made it urgent to find biodegradable substitutes. Poly(l-lactic acid) (PLLA) as a biodegradable aliphatic polyester derived from biomass resources has attracted intensive attention and already been applied in many fields such as medicine and packaging. To further expand its application range, however, much more efforts are still required to overcome some shortcomings including low crystallization rate, low toughness and poor heat resistance [1,2].

Many works aimed at improving the performances of PLLA have been reported. Among these, blending is a favorable method due to low cost and simple operation. Some works involve blending nucleating agents into PLLA to accelerate its crystallization rate because the degree of crystallinity, crystal size and morphology significantly influence the microstructure of materials and cause changes in mechanical and thermal properties [3,4,5,6]. While some other works are focused on blending PLLA with flexible polymers, flexible biodegradable polyesters, such as poly(ε-caprolactone) (PCL) [7,8], poly(ethylene succinate) (PES) [9], poly(butylene succinate) (PBS) [10], poly(butylene succinate-*co-*ethylene succinate) (PBES) [11] and poly(butylene succinate-*co*-l-lactate) (PBSL) [2], are especially preferential because the degradability of resulting blend materials could be fully maintained. In addition, blends composed of PLLA and PBS-based copolyesters captured our special attention because the introduction of many PBS-based copolyesters has been reported to be capable of not only notably improving the ductility, but also facilitating the crystallization of PLLA. For example, Yamaguchi and Yokohara reported that low content of PBS or PBSL (5–20 wt %) could homogeneously disperse as 0.1–0.4 μm particles in PLLA matrices and increase elongation at breaks of PLLA [2]. Jiao et al. prepared PLLA/PBES blend films with various compositions via the solution casting method. They found that the crystallization rate of PLLA increased with increasing PBES content, and elongation at break increased from 10% for PLLA to 455% for blends with 80 wt % of PBES. However, these studies also concluded that the blends are immiscible [11], and the interfacial tension of PLLA/PBS blend was evaluated as 3.5 mN/m [10].

The extent of miscibility between blending components affects the phase separation structure such as size and number of particles, and thus macro-properties [10]. Therefore, enhancing the miscibility of PLLA with guest polymers is important for further improvement of properties. In recent studies, twice functionalized nanoclay (TFC) was synthesized by reacting silane compound with hydroxyl groups of organoclay to endow it with additional functional groups, and was subsequently used as an in situ reactive compatibiliazer for PLLA/PBS blends. The obtained PLLA/PBS/TFC nanocomposites exhibited elevated tensile strength, tensile modulus and elongation at break as well as thermal stability. This was originated from increased interfacial interaction through chemical reaction between epoxy group of TFC and functional groups of PLLA/PBS [12,13]. Moreover, intermolecular hydrogen bonding interaction, formed between inherent hydroxyl group PVPh and carbonyl group of various polyesters, is also adopted to fabricate miscible polymer blends, including PBS/PVPh [14,15,16], PLLA/PVPh [17], PHB/PVPh [18], etc. In this work, intermolecular hydrogen bonding is intentionally introduced into poly(butylene succinate-*co*-butylene adipate) (PBSA)/PLLA blends to improve its miscibility by incorporating PVPh, which acts as a compatibilizer. The crystallization behavior, morphology and crystal structure of PBSA/PVPh/PLLA blends are systematically investigated.

## 2. Materials and Methods

### 2.1. Materials

PLLA resins were purchased from Zhejiang Hisun Biomaterials Co., Ltd. (Taizhou, China). The *M*_w_ and *M*_n_ determined by gel permeation chromatography (GPC) were 1.0 × 10^5^ and 5.8 × 10^4^, respectively. PBSA with 5 mol % adipate monomers was purchased from Anqing Hexing Chemicals Co., Ltd. (Anqing, China), *M*_w_ and *M*_n_ were 9.5 × 10^4^ and *M*_n_ = 4.8 × 10^4^, respectively. PVPh was purchased from Sigma Aldrich (St. Louis, MO, USA). These polymers were dried prior to use to remove residual moisture. *N*,*N*′-dimethyl formamide (DMF) and tetrahydrofuran (THF) were supplied from Tianjin Fuyu Fine Chemical Co., Ltd. (Tianjin, China).

### 2.2. Sample Preparation

PBSA/PVPh/PLLA ternary blends were prepared via a solution casting method using DMF as a mutual solvent. The weight ratio of PLLA to PBSA was maintained 1:1. PVPh was weighed accurately and dissolved into DMF solutions of PBSA/PLLA to obtain ternary blend samples with different PVPh content. The polymer solutions were stirred at 70 °C for 4 h and then casted into Petri dishes. Subsequently, the polymer solutions were heated appropriately with infrared light in a fume cupboard to evaporate DMF. The resulting blend films were further dried in a vacuum and collected for characterization. The samples are denoted as PVPh-*x* below to represent blend sample containing *x* wt % of PVPh.

### 2.3. Fourier Transform Infrared Spectroscopy (FTIR) Characterization

Solutions with ca. 1 wt % polymer concentration were dropped onto the surfaces of KBr wafers for FTIR testing. Solvent was allowed to evaporate and was further dried under vacuum for 12 h. An FTIR test was performed with a resolution of 4 cm^−1^ in the range of 4000–400 cm^−1^, using a blank KBr pallet as the background.

### 2.4. Differential Scanning Calorimetry (DSC)

Crystallization and melting behavior analysis of blend samples were carried out on a TA Q20 differential scanning calorimeter (TA Instruments, New Castle, DE, USA) in nitrogen atmosphere. The blend samples were encapsulated in aluminium pans, heated to 200 °C and maintained for 3 min to erase any thermal history. For non-isothermal crystallization, the samples were cooled to 0 °C, followed by a second heating scan at a rate of 10 °C/min. The isothermal crystallization was examined by a two-step procedure. Molten state samples were cooled at avmaximum rate to preselected temperatures (118, 120, 122, 124 °C) and held long enough to allow complete crystallization of PLLA, and then quenched at lower temperatures (64, 66, 68, 70 °C) and maintained until heat flow did not vary with time to crystallize PBSA.

### 2.5. Scanning Electron Microscope (SEM)

All samples were etched in THF at room temperature for 12 h to dissolve PVPh for higher contrast. The films were then dried and fractured. The fracture surfaces were used for observation with a Hitachi 5-3400 scanning electron microscope (Hitachi, Tokyo, Japan) after sputter coated with gold.

### 2.6. Wide Angle X-ray Diffraction (WAXD)

WAXD patterns for PBSA/PVPh/PLLA blends were characterized using a Rigaku D/max2000PC diffractometer (Rigaku, Tokyo, Japan) with Cu K_α_ radiation (λ = 0.154 nm). The tube voltage and current were 40 kV and 30 mA, respectively.

## 3. Results

### 3.1. FTIR Analysis

It had already been reported that the hydroxyl groups of PVPh had the capability to form intermolecular hydrogen bonding with carbonyl groups of PBSA and PLLA in binary PVPh/PLLA and PVPh/PBSA blends, respectively [14,15,16,17]. Combined with the chemical structure of these polymers, it would be reasonable to anticipate that the carbonyl groups of PBSA and PLLA will form intermolecular hydrogen bonding interaction with hydroxyl groups of PVPh. To verify the intermolecular hydrogen bonding in PBSA/PVLh/PLLA blends, the FTIR spectra of samples in the carbonyl stretching vibration region were analyzed using a peak fitting program (Peakfit, V4.12, Seasolve Software Inc., San Jose, CA, USA). This program detects hidden peaks by second derivative minima. It adjusts the peak location, line shape, peak width and height based on a least-squares parameter-adjustment criterion using the Gaussian amplitude function to obtain the best fit [19,20].

**Figure 1:**
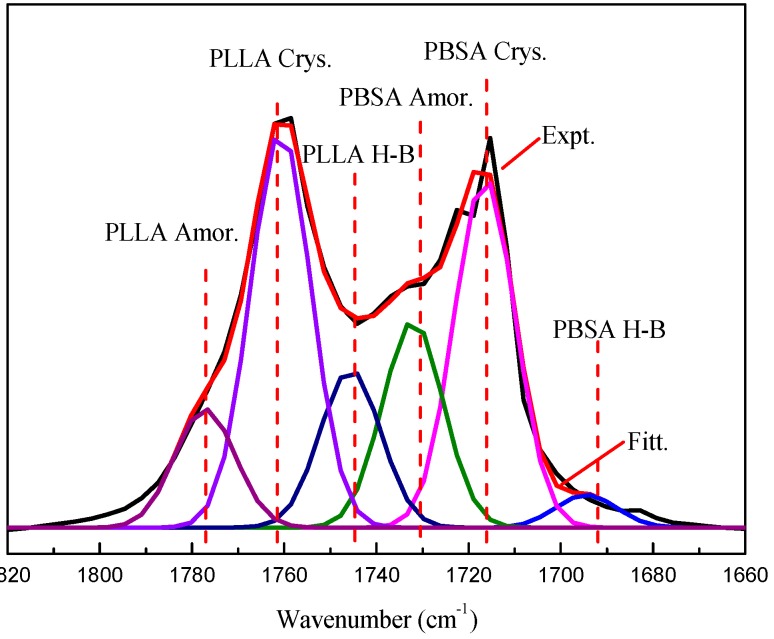
Curve fitting result for PVPh-10 (10 wt % poly(vinyl phenol)) in carbonyl stretching vibration as a typical example. Expt.: experimental observed spectrum; Fitt.: Fitted spectra; PLLA (poly(l-lactic acid)) Amor.: amorphous component in PLLA; PLLA Crys.: crystalline component in PLLA; PLLA H-B: H-bonded carbonyl groups in PLLA; PBSA (poly(butylene succinate-*co*-butylene adipate)) Amor.: amorphous component in PBSA; PBSA Crys.: crystalline component in PBSA; and PBSA H-B: H-bonded carbonyl groups in PBSA.

The curve fitting result for PVPh-10 in the carbonyl stretching vibration region is shown in Figure 1 as an example. It can be seen that the fitted spectrum is highly coincident with the experimentally obtained one, proving that this peak fitting program is applicable. The two strong absorption bands at 1760 and 1718 cm^−1^ are attributed to carbonyl stretching vibration in PLLA and PBSA, respectively. The band at 1760 cm^−1^ is resolved into three hidden peaks at 1777, 1760 and 1745 cm^−1^, which are ascribed to amorphous, crystalline and hydrogen bonded carbonyl vibration absorption in PLLA chains [21]. The band at 1718 cm^−1^ is also resolved into three hidden peaks at 1732, 1716 and 1695 cm^−1^, which are ascribed to amorphous, crystalline and hydrogen bonded carbonyl vibration absorption in PBSA chains [20]. The area of each hidden peaks were also derived to estimate the fraction of amorphous, crystalline and hydrogen bonded carbonyl groups in PLLA and PBSA chains. That is, *f*_i_ = *A*_i_/∑*A*, where *f*_i_ is the fraction of component i, A_i_ is the area of resolved hidden peak corresponding to component i, and ∑*A* is the total peak area. The estimated fractions of each component in blend samples are listed in Table 1. It is interesting to find that the fraction of hydrogen bonded carbonyl groups in PLLA fluctuates in the small range of 12.0%–13.2%, while that in PBSA increases from 2.6%–4.0% with increasing PVPh content.

### 3.2. Miscibility of PBSA/PVPh/PLLA Blends

**Figure 2:**
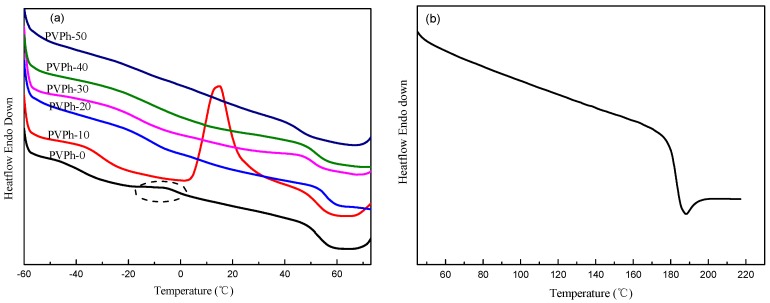
Differential scanning calorimetry (DSC) heating curves for melt-quenched (**a**) PBSA/PVPh/PLLA blends and (**b**) PVPh at a rate of 10 °C/min.

The miscibility of the blending components in an amorphous state can be assessed based on glass transition behavior of blends, provided that the difference in *T*_g_s of the two components is more than 20 °C [22]. A fully miscible polymer blend exhibits only one *T*_g_, whereas completely immiscible blends usually have two independent *T*_g_s close to those of the individual component. If the *T*_g_s of components shift to each other after blending, the two components are partially miscible. Figure 2 shows the DSC heating curves for melt-quenched samples. As can be seen from the DSC curve for PVPh-0, the *T*_g_s for PBSA and PLLA in blends were around −37.7 °C at 51.6 °C, respectively, which are close to those of neat components [19,20,23]. This suggests that PBSA and PLLA are completely immiscible in the absence of PVPh, which is in accordance with that reported in literature [10]. PVPh is an amorphous polymer with high glass transition temperature of about 183 °C as shown in Figure 2b. The *T*_g_s for PBSA increased with PVPh addition, while the *T*_g_s for PLLA remained almost constant. The approaching *T*_g_s indicate that the miscibility of PBSA and PLLA were improved. When 50 wt % of PVPh was incorporated into a PBSA/PLLA blend, only one single *T*_g_ around 45.0 °C could be observed, implying that PBSA and PLLA are miscible in an amorphous state. The *T*_g1_ for PBSA and *T*_g2_ for PLLA are listed in Table 2.

It is also interesting to note that a weak exothermic peak around −6.0 °C and a much stronger one at 18.0 °C were present on DSC curves for PVPh-0 and PVPh-10, respectively, which could be attributed to cold crystallization of PBSA component in blends. However, no discernible peaks were observed at corresponding locations for samples with higher PVPh content. These can be explained that the introduction of PVPh retarded crystallization of PBSA. The crystallization rate of PBSA is too fast in PVPh-0, so it crystallized to a relatively high crystallinity degree during the rapid cooling process. Remaining crystallizable segments completed crystallization following the heating process at a rate of 10 °C/min, which formed a weak exothermic peak on the DSC curve. For PBSA in PVPh-10, the crystallization was somewhat impeded due to intermolecular hydrogen bonding formation and did not crystallize or crystallized very little after rapid cooling, resulting in a highly amorphous state. Cold crystallization occurred during following heating process and formed a stronger exothermic peak in a higher temperature range than those of PBSA in PVPh-0. With further PVPh addition, the crystallization of PBSA in blend samples was largely hindered and did not crystallize in rapid cooling and subsequent heating process.

**Figure 3:**
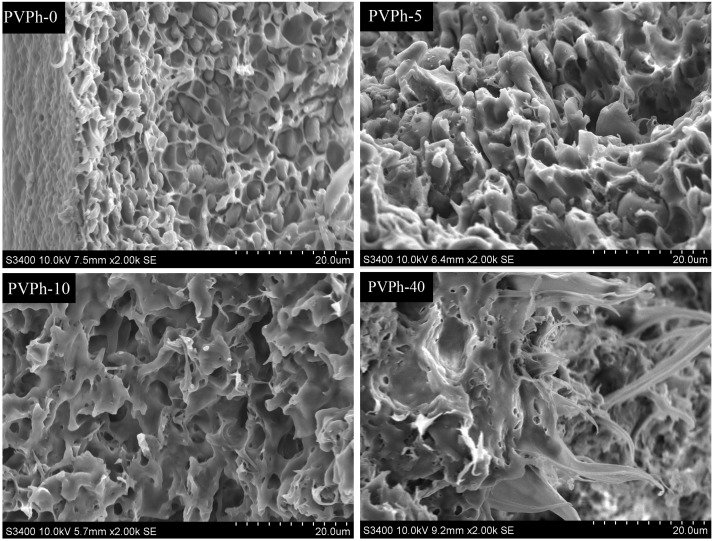
Scanning Electron Microscope (SEM) micrographs for PBSA/PVPh/PLLA fracture surfaces after being etched with tetrahydrofuran.

SEM micrographs can provide further information about miscibility and microstructure of blend samples. Figure 3 shows the fracture surface morphologies for PBSA/PVPh/PLLA blends. A typical “sea-island” phase separation structure was observed in SEM photographs for blend without PVPh incorporation, which means poor miscibility between PBSA and PLLA. With 5 wt % PVPh incorporation, however, the morphologies of PBSA/PVPh/PLLA blends moved into the homogenous phase. Such an effect became more evident when PVPh content further increased to 10 wt % and above, for example, 40 wt % as presented in Figure 3. These results again verify that the miscibility between PBSA and PLLA was improved. In addition, more ductile fractures were observed for samples with higher PVPh content compared with those without PVPh incorporation, which suggests that the addition of PVPh may result in better toughness or ductility.

### 3.3. Non-Isothermal Crystallization and Melting Behavior of PBSA/PVPh/PLLA Blends

**Figure 4:**
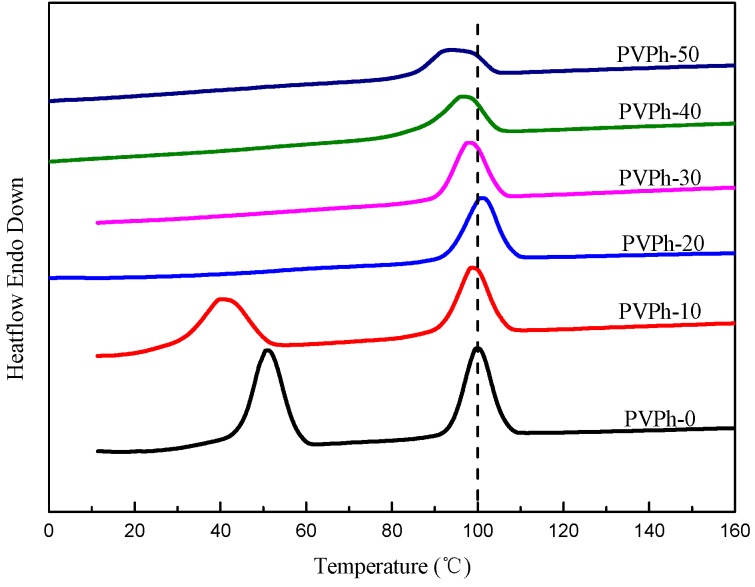
Non-isothermal crystallization DSC curves for PBSA/PVPh/PLLA blends.

Non-isothermal crystallization DSC curves for PBSA/PVPh/PLLA blends are presented in Figure 4. The non-isothermal crystallization parameters are listed in Table 2. As can be seen from Figure 4, the crystallization peaks of PBSA (*T*_c1_) and PLLA (*T*_c2_) are around 50.9 °C with a Δ*H*_c1_ of 21.7 J/g and 99.9 °C with a Δ*H*_c2_ of 16.8 J/g for PVPh-0, respectively, which are close to those of neat components [20,23]. The crystallization peak for PBSA shifts to 40.1 °C with a Δ*H*_c1_ of 16.6 J/g when 10 wt % PVPh was added into PBSA/PLLA blends. With further PVPh incorporation, however, the crystallization peaks for PBSA disappear on cooling DSC curves, implying that the crystallization of PBSA was completely impeded due to the formation of intermolecular hydrogen bonding and the dilution effect [14,15,16]. *T*_c2_ almost remains constant in blends with 20 wt % and less PVPh incorporation as shown in Figure 4. When further increasing PVPh content, *T*_c2_ decreases within a narrow range. The crystallization enthalpy of PLLA decreases with PVPh addition as presented in Table 2.

### 3.4. AvramiAnalyses for Isothermal Crystallization of PBSA/PVPh/PLLA Blends

**Figure 5:**
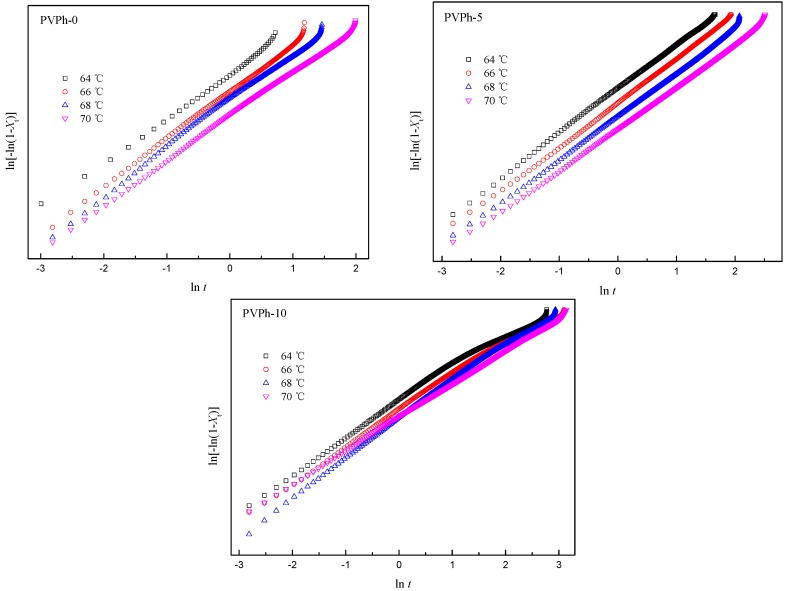
Avrami plots for PBSA isothermally crystallization in blend samples.

PVPh-0, PVPh-5 and PVPh-10 were selected for isothermal crystallization analysis because samples with higher PVPh content crystallized too slowly or did not crystallize as a result of hydrogen bonding formation. The Avrami equation was employed to analyze the isothermal crystallization of blend samples with 0, 5 and 10 wt % of PVPh in the following form [24]:

(1) $$1-X_{t}=\exp(-kt^{n}),$$

where *X_t_* is the relative crystallinity degree at time *t*, *k* is the crystallization rate constant and *n* is the Avrami exponent dependent on crystallization mechanism and crystal growth dimensions. Using the double logarithmic form of Equation (1), one can obtain *k* and *n* values from slopes and intercepts of plots of ln[−ln(1 − *X_t_*)] versus ln*t*, respectively. The Avrami plots for isothermal crystallization of PBSA in blend samples at designated temperatures are presented in Figure 5. As can be seen, the plots of ln[−ln(1 − *X_t_*)] against ln*t* generally show good linear correlation, which means that the Avrami theory was applicable in the isothermal crystallization of PBSA in PBSA/PVPh/PLLA blends. However, it is also noticeable that deviations from Avrami plots occur in the later stages of crystallization, which is ascribed to spherulite impingement [20,25]. The Avrami parameters were obtained using the linear portion of plots of ln[−ln(1 − *X_t_*)] versus ln*t* and are listed in Table 3. Avrami exponent *n* values listed in Table 3 were decimals between 2.3–2.7, indicating that crystalline branching and/or two stage crystal growth might present in the crystallization process, and the PBSA spherulites assumed a two-dimensional to three-dimensional growth with a combination of homogeneous and heterogeneous nucleation [20].

The crystallization half time (*t*_1/2_) was derived from the following equation to investigate effects of PVPh addition on the crystallization rate:

(2) $$t_{1/2}={\left(\ln2/k\right)}^{1/n}.$$

The crystallization half time of PBSA (*t*_1/2_) isothermally crystallized at designated temperatures (*T*_c_s) presented in Table 3 increases notably with weight fraction of PVPh and *T*_c_s, which means that the addition of PVPh decreases the crystallization rate of PBSA in PBSA/PVPh/PLLA blends.

**Figure 6:**
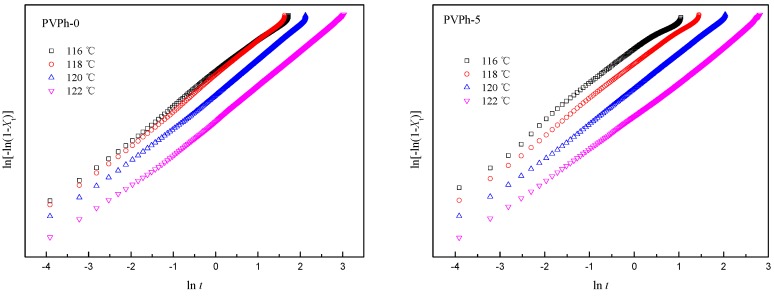

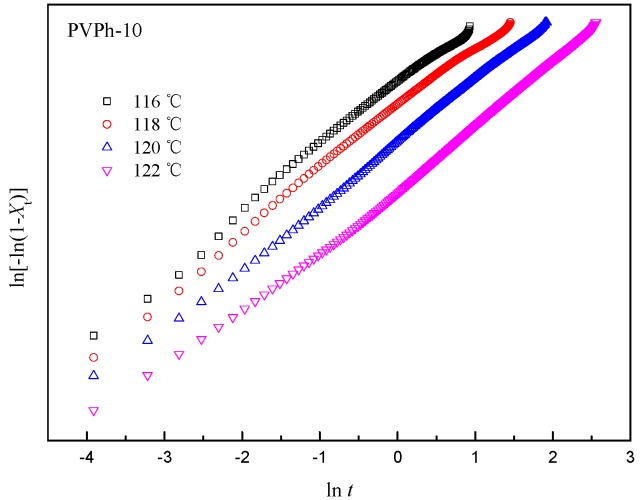
Avrami plots for PLLA isothermally crystallization in blend samples.

Avrami analyses for isothermal crystallization of PLLA in blend samples were performed using similar methods. The Avrami plots for PLLA isothermal crystallization in blend samples are shown in Figure 6. Deviations in the later stages of crystallization are also interpreted as spherulite impingement [20,25]. Avrami parameters for PLLA isothermal crystallization in blend samples are presented in Table 4. The values of Avrami exponent *n* for PLLA isothermal crystallization are between 2.6–2.8 and very close to 3, implying that the PLLA spherulites assumed three-dimensional growth with heterogeneous nucleation [19]. *t*_1/2_ for PLLA isothermally crystallized at indicated *T*_c_s listed in Table 4 decreases slightly with PVPh addition, which means that the crystallization rate of PLLA was also increased.

### 3.5. WAXD Analysis

**Figure 7:**
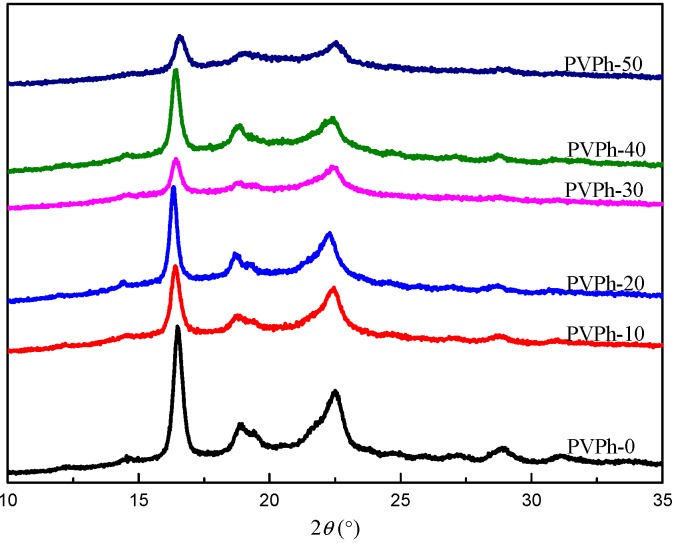
WAXD patterns for PBSA/PVPh/PLLA blend samples.

WAXD patterns for PBSA/PVPh/PLLA blend samples are shown in Figure 7. The diffraction peaks at 2θ = 16.5°, 18.8°, 28.8° are attributed to (110), (111), (300) crystallographic plane of PLLA crystal in *α*′ form, respectively [26,27]. However, those at 2θ = 19.4°, 21.9°, 22.5° are ascribed to (020), (021), (110) crystallographic plane of PBSA, respectively [23]. The diffraction peaks for PLLA remained relatively sharp for samples with all investigated PVPh content, suggesting that PLLA was partly crystallized. However, the diffraction peaks for PBSA vanished gradually with increasing PVPh content, indicating that PBSA turned into a fully amorphous state when introducing high content of PVPh into PBSA/PLLA blends. These results are in accordance with preceding non-isothermal crystallization analysis. This is a very interesting phenomenon because it seems that PVPh in blends “chooses” to interact more with PBSA than with PLLA. However, investigations about possible reasons are beyond the scope of this work.

## 4. Conclusions

PVPh was introduced into PBSA/PLLA blends via solution casting to improve the miscibility between biodegradable PBSA and PLLA. It was proved by FTIR analysis that PVPh had formed intermolecular hydrogen bonding with PBSA and PLLA, which acted as crosslinking agents between PBSA and PLLA. The miscibility between PBSA and PLLA were improved with PVPh introduction evidenced by approaching *T*_g_s of the two components in DSC analyses. When PVPh content reached up to 50 wt %, the blend sample exhibited only one *T*_g_, meaning complete miscibility between PBSA and PLLA. The improved miscibility of PBSA/PLLA blends was further confirmed by SEM. Typical “sea-island” phase separation structure for PBSA/PLLA blends transformed into one phase structure for PBSA/PVPh/PLLA blend samples. In addition, the melt crystallization peak of PBSA in blend samples shifted to lower temperatures with PVPh addition and vanished when adding 20 wt % PVPh and above. PLLA also shows a decreasing trend. Avrami analyses suggested that crystallization of PBSA in blend samples assumed two- to three-dimensional crystal growth with a combination of homogeneous and heterogeneous nucleation, while that of PLLA assumed three-dimensional crystal growth with homogeneous nucleation. The crystallization rate of PBSA was distinctly decreased by PVPh incorporation. On the contrary, the crystallization rate of PLLA increased slightly with PVPh addition. WAXD analysis indicated that PLLA in blend samples remained partly crystallized, while PBSA turned into an amorphous state with increasing PVPh content.

## Figures and Tables

**Table 1:** Estimated fractional values for amorphous, crystalline and H-bonded carbonyl groups in PLLA and PBSA.

Samples	PLLA			PBSA		
	*f*_Amor._ (%)	*f*_Crys._ (%)	*f*_H-B_ (%)	*f*_Amor._ (%)	*f*_Crys._ (%)	*f*_H-B_ (%)
PVPh-10	9.40	31.43	12.51	16.37	27.68	2.61
PVPh-20	9.70	31.34	12.09	16.34	26.77	3.79
PVPh-40	8.53	31.28	13.26	16.61	26.32	4.00

**Table 2:** *T*_g_ and non-isothermal crystallization DSC data for PBSA/PVPh/PLLA blends.

Samples	*T*_c1_ (°C)	Δ*H*_c1_ (J/g)	*T*_c2_ (°C)	Δ*H*_c2_ (J/g)	*T*_g1_ (°C)	*T*_g2_ (°C)
PVPh-0	50.91	21.68	99.91	16.82	−37.69	51.58
PVPh-10	40.11	16.55	98.57	15.23	−30.40	52.36
PVPh-20	-	-	100.78	14.37	−11.08	56.04
PVPh-30	-	-	97.70	12.37	−15.15	51.61
PVPh-40	-	-	96.24	11.86	−11.26	51.92
PVPh-50	-	-	93.32	8.85	45.04	45.04
PVPh-100	-	-	-	-	-	182.98

**Table 3:** Avrami parameters for PBSA isothermal crystallization in blend samples.

Samples	*T*_c_ (°C)	*n*	*k* (min^−n^)	*t*_1/2_ (min)
PVPh-0	64	2.4	5.4 × 10^−1^	1.11
	66	2.5	2.0 × 10^−1^	1.63
	68	2.5	0.7 × 10^−2^	2.58
	70	2.3	0.6 × 10^−2^	2.96
PVPh-5	64	2.7	9.6 × 10^−2^	2.07
	66	2.7	4.0 × 10^−2^	2.83
	68	2.7	1.7 × 10^−2^	3.90
	70	2.6	0.8 × 10^−3^	5.56
PVPh-10	64	2.3	0.1 × 10^−2^	5.92
	66	2.3	0.7 × 10^−3^	7.59
	68	2.7	0.3 × 10^−3^	7.66
	70	2.4	0.3 × 10^−3^	9.18

**Table 4:** Avrami parameters for PLLA isothermally crystallization in blend samples.

Samples	*T*_c_ (°C)	*n*	*k* (min^−n^)	*t*_1/2_ (min)
PVPh-0	116	2.6	7.8 × 10^−2^	2.30
	118	2.8	6.4 × 10^−2^	2.32
	120	2.8	1.5 × 10^−2^	3.93
	122	2.8	1.7 × 10^−2^	8.56
PVPh-5	116	2.6	4.8 × 10^−1^	1.15
	118	2.7	1.7 × 10^−1^	1.71
	120	2.8	2.7 × 10^−2^	3.24
	122	2.8	2.7 × 10^−3^	7.37
PVPh-10	116	2.7	0.6 × 10^−1^	1.04
	118	2.6	0.2 × 10^−1^	1.55
	120	2.7	5.1 × 10^−2^	2.62
	122	2.8	6.4 × 10^−3^	5.40
